# Intraspecific Competition Promotes Oviposition During Host Egg Incubation in the Parasitic Fly *Philornis downsi*

**DOI:** 10.1002/ece3.71892

**Published:** 2025-08-03

**Authors:** Barbara Kofler, Denis Mosquera, George E. Heimpel, Heinz Richner, Sabine Tebbich

**Affiliations:** ^1^ Department of Behavioural Biology and Cognition University of Vienna Vienna Austria; ^2^ Charles Darwin Foundation Puerto Ayora Ecuador; ^3^ Universidad Técnica de Machala Machala Ecuador; ^4^ Department of Entomology University of Minnesota St. Paul Minnesota USA; ^5^ Instituto Nacional de Biodiversidad (INABIO) Quito Ecuador; ^6^ Department of Biology University of Bern Bern Switzerland

**Keywords:** Darwin's finches, density dependence, host–parasite interaction, intraspecific competition, life history, *Philornis downsi*

## Abstract

Avian biodiversity declines are increasingly linked to invasive parasites threatening local bird populations that lack evolved defenses. The level of virulence in these interactions, in turn, influences the parasites' own fitness and drives co‐evolutionary dynamics. Studying newly established host–parasite systems can shed light on adaptive processes and associated behavioral and ecological aspects. The invasive parasitic fly *Philornis downsi*, unintentionally introduced to the Galapagos Islands, severely threatens native bird species, including endemic Darwin's finches. Over the past decades, *P. downsi* infestation rates have increased, shortening nestling survival and limiting larval development. Furthermore, an earlier shift in oviposition timing has been documented, with female flies infesting incubated host nests, despite *P. downsi* having previously been classified as an obligate nestling parasite. In this study, we investigated the hypothesis that intraspecific competition, shaped by host density and parasite infestation patterns, drives oviposition during the incubation of bird eggs in *P. downsi*. Host density, interacting with mean infestation intensity of simultaneously active nests, positively influenced oviposition during host incubation, and long‐term data identified the proportion of infested nests (prevalence) as a key driver. When mean infestation intensity was low, higher host density increased per‐nestling infestation and reduced larval survival, suggesting clustered nests attract more flies. In contrast, under high mean infestation intensity, greater host density led to lower per‐nestling infestation and higher larval survival. Our findings suggest high fly competition drives the earlier shift in the parasite's life cycle, while associated trade‐offs or potential adaptive strategies may explain the observed decrease in per‐nestling infestation intensity. Moreover, oviposition during host incubation was not observed in mainland Ecuador, the native range of the ancestral *P. downsi* population. Despite higher *Philornis* species diversity, reduced competition in mainland Ecuador contrasts with high infestation rates in Galapagos, indicating rapid behavioral divergence due to increased competition in the latter location.

## Graphical abstract

This study investigates the mechanisms driving an earlier shift in oviposition timing of *Philornis downsi*, an invasive parasitic fly threatening native Galapagos bird species, including endemic Darwin's finches. While *P. downsi* was historically classified as an obligate nestling parasite, recent observations in Galapagos have revealed that females now oviposit during host egg incubation. Our study identified intraspecific competition—shaped by host density and infestation patterns—as a key driver of oviposition during host incubation in *P. downsi*.

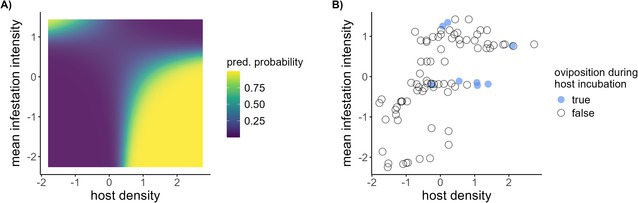

## 1 Introduction

Declines in avian biodiversity are often caused by invasions or unintentional introductions of bird parasites (Atkinson and LaPointe 2009), a problem that is on the rise due to the expansion of the global network of pathways (Tourchin et al. 2002; Westphal et al. 2008). In the absence of natural enemies and competitors, alien parasites can exert devastating effects on naïve host communities lacking co‐evolved resistance or tolerance (Allison 1982; Fassbinder‐Orth et al. 2013; Lymbery et al. 2014). However, parasite fitness, in return, depends on the successful reproduction in a host (Paterson and Piertney 2011; Tschirren et al. 2007). In this context, mechanisms of natural selection may drive co‐evolutionary dynamics (Gandon et al. 2008) wherein parasite adaptations to successfully exploit host resources are countered by adaptations of hosts to reduce the effects of parasitism (Ebert and Weisser 1997; Ewald 1995). From an evolutionary perspective, understanding the trade‐off between the benefits to the parasite and the costs to the host is central for identifying selective mechanisms at work (Ebert and Bull 2008). Newly established host–parasite interactions in natural ecosystems are ideal for revealing adaptive processes, as they allow the evaluation of host–parasite dynamics at early stages of interaction and provide the rare opportunity to investigate behavioral, ecological, and genetic aspects of these complex relationships in the wild.

Such a novel model host–parasite system is found on the Galapagos archipelago, where the unintentionally introduced parasitic fly *Philornis downsi* Dodge & Aitken (Diptera: Muscidae) meets a naïve and isolated host community. As a generalist bird parasite, *P. downsi* has a broad host range (Bulgarella et al. 2015; Bulgarella and Heimpel 2015; Löwenberg‐Neto 2008) that may have facilitated the successful establishment of novel host–parasite relationships by the fly in Galapagos and its wide spread across the archipelago after its introduction in the decades preceding the 1960s (Basnet et al. 2025; Causton et al. 2006). In Galapagos, *P. downsi* is known to infest nearly all small altricial landbirds (Common, Dudaniec, et al. 2020; McNew and Clayton 2018), including 12 of 17 species of Darwin's finches (Fessl et al. 2018; S. A. Knutie, personal communication).

Adult flies are free‐ranging and non‐parasitic (Fessl et al. 2018). Up to six female flies lay their eggs into host nests (Dudaniec et al. 2010), and the duration of the egg stage can be highly variable. The time from oviposition to egg hatch can be as short as 6–24 h (Causton et al. 2011; Kleindorfer et al. 2014; Lahuatte et al. 2016), depending in part on temperature and humidity (Sage et al. 2018). The first larval stage is usually found in the nares and/or auditory canals of young finch nestlings. After their development into 2nd and 3rd instar larvae, they move to the nest base, where they emerge during nighttime to externally feed on nestling blood and tissue by penetrating their skin (Fessl, Kleindorfer, and Tebbich 2006).

In Galapagos, *P. downsi* parasitism causes high levels of nestling mortality (Dudaniec et al. 2010; Fessl, Sinclair, and Kleindorfer 2006; Kleindorfer and Dudaniec 2016) and is thus considered to be one of the biggest threats to the survival of populations and species of Darwin's finches and other landbirds (Causton et al. 2011; Fessl et al. 2018). Both parasite prevalence (the proportion of potential host nests containing *P. downsi*) and infestation intensity (the number of *P. downsi* larvae per infested nest) reach high levels on the islands compared to mainland Ecuador. In mainland Ecuador, only about 30% of passerine bird nests are parasitized by *P. downsi*, with an average of approximately 10 larvae per infested nest (Bulgarella et al. 2017; Ramirez et al. 2022). In contrast, nearly 100% of finch nests with hatched nestlings in Galapagos are infested, often harboring over 30 and sometimes more than 100 larvae (Bulgarella et al. 2015; Cimadom et al. 2014; Dudaniec et al. 2007; Heimpel et al. 2017; Kleindorfer and Dudaniec 2016). The interaction between *P. downsi* and its hosts on the continent is much older than that on Galapagos, suggesting that an evolutionary equilibrium has been reached.

When a parasite's fitness hinges on the accessibility of a finite and unevenly distributed host resource, density‐dependent growth or survivorship patterns may arise within its population due to intraspecific competition (Tripet and Richner 1999). Consequently, competition is among the key factors shaping the evolution of virulence, as it impacts parasite population dynamics and natural selection. In this context, fly intraspecific competition may contribute to the dynamics between *P. downsi* and its hosts in Galapagos, as evidenced by the significant increase in larval abundance per nest and the trend toward earlier and more synchronous oviposition during the nesting cycle observed over the past decades (Kleindorfer et al. 2014). This is associated with fitness consequences for the host, as young nestlings are exposed to a higher number of large and older larvae, which results in significantly shorter survival periods (Cimadom and Tebbich 2021; Kleindorfer et al. 2014; Kleindorfer and Dudaniec 2016). However, the increase in infestation intensity also comes at a cost to the parasite. The narrowing time window of host resource availability negatively affects pupation success, adult fly body size, and fecundity (Common, O'Connor, et al. 2020; Honěk 1993; Kleindorfer et al. 2014). This time constraint and host limitation likely intensify intraspecific competition, either among female flies competing for suitable host nests or among larvae within nests, potentially creating strong selection pressures for traits that enhance the parasite's ability to effectively exploit hosts and optimize reproductive strategies.

Aligned with this, the fly's oviposition timing has advanced even further in the host's nesting cycle in the recent past. In 2012, larvae were first observed in nests containing only host eggs, where no hatched nestlings have ever been present (Cimadom et al. 2016). This suggests a change in the fly's oviposition tactic, with larvae now either feeding on incubating female birds (Common, Dudaniec, et al. 2020) or “waiting” to feed on hatchlings (Sage et al. 2018). Moreover, parasite prevalence during the incubation phase has been found to differ between two sympatric Darwin's finch species, with a higher percentage of infested nests during incubation in the Green Warbler Finch ( *Certhidea olivacea* ) compared to the Small Tree Finch ( *Camarhynchus parvulus* ) (Cimadom and Tebbich 2021).

Time‐series analyses have shown that the population size of *P. downsi* females appears stable and that it is primarily regulated by direct density dependence (Causton et al. 2019). In Darwin's finches, density‐dependent infestation patterns were not only found to vary temporally (in the course of the breeding season), but also spatially due to environmental heterogeneity (i.e., host breeding density), with more larvae observed in aggregated host nests (Kleindorfer and Dudaniec 2009). Therefore, we hypothesize that density‐dependent competition among female flies, shaped by spatiotemporal variations in host density and overall fly abundance, drives oviposition during host incubation.

This study investigates the role of competition among flies in driving the earlier shift in the life cycle of *P. downsi* in two different host species, the Green Warbler Finch and the Small Tree Finch. To evaluate the competition hypothesis, we assessed whether levels of host density and mean infestation intensity of simultaneously active nests are associated with oviposition during host incubation. Additionally, we investigated how these factors affect per‐nestling infestation intensity, the duration of nest activity, and the reproductive success of flies at the level of individual nests. Under the competition hypothesis, we predicted that both infestation patterns and the probability of infestation during incubation are influenced by both host density and mean infestation intensity, with a greater number of nests and spatial clustering leading to higher per‐nestling infestation intensity and higher likelihood of *P. downsi* ovipositing during host incubation. We further hypothesized that species‐specific differences in oviposition timing associated with the two finch species result from spatial and/or temporal heterogeneity in species distributions. Consequently, we investigated species‐specific variations in host density at nest sites.

We also used a long‐term dataset to assess whether oviposition during host incubation could be predicted by overall parasite infestation patterns (intensity and prevalence) as proxies for fly competition. We hypothesized a positive association between both predictors and oviposition during host incubation.

Moreover, to better contextualize key factors contributing to a hypothesized rapid evolution of fly life‐history traits in Galapagos, we assessed the occurrence of oviposition during host incubation and infestation patterns in the ancestral population on mainland Ecuador.

## 2 Materials and Methods

### 2.1 Study Site

The study was conducted near “Los Gemelos” in the humid highland region of Santa Cruz Island, Galapagos (0°37′50″ S, 90°23′25″ W, 569–634 m a.s.l.). The study site is part of a protected area within the Galapagos National Park, primarily vegetated by *Scalesia pedunculata* forest.

The *Scalesia* zone on Santa Cruz harbors the island's highest density of arboreal Darwin's finches (Cimadom et al. 2019; Dvorak et al. 2012) and the passerine community at our study site predominantly includes the Green Warbler Finch, Small Tree Finch, Small Ground Finch ( *Geospiza fuliginosa* ), Woodpecker Finch ( *Camarhynchus pallidus* ), and Yellow Warbler ( *Setophaga petechia* ). All of these species belong to the host spectrum of *P. downsi*.

### 2.2 Nest Monitoring and *P. downsi* Collection

Daily intensive nest searches were conducted within a 200 × 300 m plot of *Scalesia* forest during two finch breeding periods (January–March 2020 and January–April 2022), enabling nearly complete detection of active passerine nests. Following the protocol described by Cimadom et al. (2014), a total of 317 nests were monitored across both field seasons. Active nests were observed at specific time intervals based on breeding status to minimize disturbance and ensure accurate data collection. During the nest‐building phase, visits occurred every 5 days; once incubation began, nests were checked every 3 days; and after the first nestling hatched, nests were visited every other day. From the incubation stage onward, an endoscopic video camera (dnt Findoo 3.6) mounted on a lightweight carbon pole system was used for nest inspection. This enabled the determination of breeding onset, egg count, hatching day, number of nestlings, and the date of failure or fledging. Nest search and monitoring were conducted between 6:00 a.m. and 1:00 p.m.

After the cessation of nest activity was confirmed, nests were individually collected in sealed plastic bags and dismantled in the laboratory to quantify the number of *P. downsi* larvae, pupae, and empty puparia and to assess the age structure of the larval cohort. Dead nestlings found in the nests were soaked in 80% ethanol for 1–2 days to allow 1st instar larvae in their nares or ear canals to become detached and be collected. Larvae were categorized into developmental stages according to instar identification protocols (Common, Dudaniec, et al. 2020; Fessl, Sinclair, and Kleindorfer 2006). Parasite intensity was calculated as the sum of larvae, pupae, and empty puparia per nest.

### 2.3 Estimation of Host Hatching and Oviposition Dates (Main Dataset 2020 and 2022)

For all nests, the duration of nest activity (in days) from the first observed incubation to the last recorded active day as well as the hatching date of the first nestling was estimated based on detailed nest monitoring data. In Darwin's finches, incubation lasts ~14 days, and if successful, nestlings fledge approximately 14 days after hatching (Cimadom et al. 2014). Therefore, for nests in which nestlings successfully hatched, the incubation start date was defined as 14 days before the hatching date. For nests abandoned during incubation, the first observed incubation date was used as the start date. For nests discovered with hatched nestlings, the incubation start date was estimated as 28 days before fledging for successful nests. If no nestlings survived, it was estimated by subtracting (chick age + 14 days) from the date of failure. The incubation start date of all nests was converted to a numerical sequence, beginning at day 1 for the earliest nest of the two seasons (December 18, range 1–108).

Key milestones of the nesting cycle, including the hatching date of the first nestling and the fledging date of the last nestling, were estimated based on direct observations. Since nestlings hatch asynchronously and considering the 3‐day monitoring interval during incubation, the hatching date of the first nestling was calculated as follows: if all nestlings were found newly hatched, the hatching date was set at 2 days before observation; if one or more eggs remained, it was set at 1 day before. In fledged nests, the hatching day was defined as 14 days before the fledging date. The last day a nest was observed to be active was considered the last day of nest activity. If a nest was empty on the predicted fledging day, fledging success was confirmed by observing fledglings in the vicinity of the nest (Heyer et al. 2021). In these cases, the last active day was estimated as the day before, unless the fledging process was directly observed, in which case it was recorded as the day of the last observation.

Out of the 317 monitored nests, 259 were observed until the end of their nesting activity. Information on *P. downsi* infestation status was obtained for 240 nests, including those that failed during host egg incubation, of which 147 were infested. Nests with *P. downsi* infestation data during incubation were classified as focal nests (*N* = 127). Oviposition during host incubation occurred when abandoned or failed nests during incubation were found to be infested, or when post‐hatching nests contained larvae or pupae older than the nestlings.

The timing of *P. downsi* infestation in nests with hatched host eggs was estimated following the protocol described in Table 1 and relies on the following minimum developmental times: 6 h for egg hatching, 1–2 days for 2nd instar larvae (estimated based on reported durations of other developmental stages), 3–7 days for 3rd instar larvae, and 4–7 days for pupae until eclosion. Observations from wild nests report a minimum time until pupation of 4–7 days and approximately 7–10 days for the duration of the pupal stage (Causton et al. 2011; Common, Dudaniec, et al. 2020; Fessl, Sinclair, and Kleindorfer 2006; Kleindorfer et al. 2014). Fessl, Sinclair, and Kleindorfer (2006) observed 1st instar larvae in nests with 1–3 day old nestlings, 2nd instar larvae in nests with 3–6 day old nestlings, and 3rd instar larvae in nests with ≥ 3 day old nestlings. To ensure accuracy, a conservative approach was used to confirm that nests identified as infested during incubation were truly infested at that time, avoiding false positives. While using minimum developmental times may underestimate the incidence of *P. downsi* infestation in finch nests during host incubation, this strategy was chosen to prioritize confidence in identifying such infestations.

**TABLE 1:** Criteria used to categorize nests based on the presence of oviposition during host incubation, the absence of oviposition during host incubation, or exclusion from analysis, determined by the developmental stage of *Philornis downsi* larvae or pupae and the timing of nestling death or predation after hatching.

Focal nests with oviposition during host incubation	Nests with dead nestlings or predation < 3 days after hatching containing 3rd instar larvae, pupae, or empty puparia
	Nests with dead nestlings or predation < 4 days after hatching containing pupae or empty puparia
	Nests with dead nestlings or predation < 7 days after hatching containing empty puparia
Focal nests without oviposition during host incubation	Nests with 1st instar larvae ≥ day 1 after hatching
	Nests with 2nd instar larvae ≥ day 3 after hatching
	Nests with 3rd instar larvae or pupae ≥ day 8 after hatching
Nests excluded	Nests ≥ 2 days after hatching with 2nd instar larvae
	Nests 3–7 days after hatching with 3rd instar larvae
	Nests 4–7 days after hatching with pupae

Host density was calculated as the sum of the inverse distances of a focal nest to all other simultaneously active nests in the study area. This method weights closer nests more heavily than those further away, resulting in a higher density at areas with more nearby active nests. Simultaneously active nests for a focal nest were defined as those from the total of 317 monitored nests whose active period overlapped with the focal nest's start of incubation. This included all nests, even those that were not monitored until the end of activity at the end of the field seasons. As this study focused on the phenomenon of oviposition during host incubation in *P. downsi*, host availability was calculated using the full active nest period, with all nests from incubation start to the activity end considered suitable for the parasite. For distance calculations, latitude and longitude coordinates (WGS84 standard) were converted to Universal Transverse Mercator (UTM) coordinates, expressed as eastings and northings, using an online converter (www.LatLong.net; 2012–2025). Distances were calculated using the following formula:

$$\text{Distance}=\sqrt{{\left(\text{easingsNEST}-\text{eastingsFOCAL}\right)}^{2}+{\left(\text{northingsNEST}-\text{northingsFOCAL}\right)}^{2}}$$

Additionally, for each focal nest, prevalence and mean infestation intensity of simultaneously active nests with hatched nestlings and full infestation information were calculated. Prevalence was defined as the proportion of parasitized nests, and mean infestation intensity was the average number of *P. downsi* larvae, including uninfested nests. During the 2020 field season, bird nesting activity was continuous, whereas in 2022 it was briefly interrupted in February due to very dry conditions, with breeding activity resuming after rainfall.

### 2.4 Long‐Term Dataset

A long‐term analysis assessed whether overall *P. downsi* infestation patterns (intensity and prevalence) could predict oviposition during host incubation. This analysis utilized a dataset from eight field seasons (2012, 2014–2017, 2022–2024), based on nest activity data from 758 nests with infestation information and known hatching dates. Mean infestation intensity (including uninfested nests) and prevalence were calculated for nests hatching within the same 2‐week interval, starting from the earliest recorded hatching date (December 28), across all years. Nests were excluded if predated, fallen, damaged, exposed to insecticides (Permacap or permethrin), or involved in other experimental treatments. Only intervals with three or more nests with nestlings were included to ensure reliable calculations. The dataset was then filtered to include only the 170 nests that failed during incubation (i.e., abandoned with eggs) and matched the prevalence and mean intensity values for the 2‐week intervals based on the date of abandonment.

### 2.5 Mainland Dataset

To investigate the infestation pattern of *P. downsi* in continental Ecuador, in 2020 and 2022–2023, nests of various passerine bird species were searched and monitored at four sites near the Ecuadorian coast: Loma Alta (1°53′57″ S, 80°37′56″ W, 61–593 m a.s.l.), Daule (1°52′54″ S, 79°54′29″ W, 4–7 m a.s.l.), Agua Blanca (1°31′41″ S, 80°44′15″ W, 20–148 m a.s.l.), and Dos Mangas (1°49′01″ S, 80°40′48″ W, 72–193 m a.s.l.). A variety of different ecosystems (deciduous forests, humid forests, rice fields) were covered. Intensive nest search was conducted between November and March. Additionally, artificial nests consisting of nest boxes and bamboo towers were installed and monitored as reported previously (Brito Vera et al. 2022; Bulgarella et al. 2017, 2015; Ramirez et al. 2022). During the nest‐building phase, nests were visited approximately weekly. Incubating nests were checked every 3–5 days and nests with hatchlings at an interval of 2–5 days. From the incubation stage onward, nests were additionally inspected with an endoscopic video camera (dnt Findoo 3.6, Depstech WF028‐SJ). A total of 489 nests were monitored, of which 89 nests failed during the incubation phase. After the cessation of nest activity was confirmed, all nests were collected and placed in sealed plastic bags. Within 10 days from nest collection, the nests were dismantled to characterize the nest material, prevalence, and intensity of *Philornis* species. *Philornis* species were identified based on spiracular plates and slits on the posterior end of the puparia (Ramirez et al. 2022); dominant species at our field sites were *P. downsi* and *P. niger* (Bulgarella et al. 2017, 2015; Ramirez et al. 2022).

### 2.6 Statistical Analysis

For each model analyzed, a standardized procedure was followed using R version 4.3.1 (R Core Team 2023). The “fe.re.tab” custom function (provided by R. Mundry) was employed to pre‐process the data, which included checking for missing values, dummy‐coding of categorical predictors, and identifying all theoretically possible random slopes. Continuous predictors were z‐transformed, and categorical dummy‐coded predictors were manually centered for integration in generalized linear mixed models using the “glmmTMB” function (version 1.1.8) (Brooks et al. 2017). Model diagnostic assessments were conducted using the “DHARMa” package (version 0.4.6) (Hartig 2022). Collinearity among predictors was assessed by calculating variance inflation factors (VIFs) with the “vif” function from the “car” package (version 3.1.2) (Fox and Weisberg 2019). All observed VIF values were below 3, indicating no concerning collinearity (Zuur et al. 2010). Subsequently, each model was compared to a null model, excluding the test predictors, using chi‐squared ANOVA to assess whether the inclusion of all test predictors significantly improved model fit. All plots were created using R package ggplot2 (version 3.5.1) (Wickham 2016).

High intercept estimates and considerable variability in the intercept were observed across models derived from the 2020–2022 dataset. These issues may arise from factors such as insufficient representation of certain levels of the predictor variables or small sample sizes for specific combinations of predictors. To assess model stability, we employed a leave‐one‐out cross‐validation (LOO‐CV) approach (models A–D, F), refitting the models by excluding one observation at a time and recalculating the mean and standard deviation (SD) of the parameter estimates.

#### 2.6.1 Infestation Patterns

##### 2.6.1.1 Temporal Trends in Infestation Patterns and Host Dynamics

Initial host density, the number of simultaneous nests, as well as the mean *P. downsi* infestation intensity and prevalence of simultaneously active nests, were compared between years using the Mann–Whitney *U* test. A normal approximation was applied to estimate the *p*‐value, focusing exclusively on the first 29 days of each season. This period was selected based on the minimum egg‐to‐egg generation time of 29 days reported in Causton et al. (2019).

##### 2.6.1.2 Host Density and Infestation Patterns in Focal Nests

To test the hypothesis that fly competition is influenced by the interaction between host density and mean infestation intensity of simultaneously active nests, we modeled the effect of this interaction on the following response variables: (A) infestation intensity per nestling, (B) duration of nest activity (as a proportion of maximum duration), and (C) the proportion of mature *P. downsi* individuals (3rd instar larvae and pupae) at the end of nest activity in focal nests. Upon the host's death, only larvae that have reached the 3rd instar stage are capable of pupating and eventually emerging as adult flies; thus, this value serves as a proxy for the reproductive success of the parasite (Cimadom and Tebbich 2021; Common et al. 2021; Kleindorfer et al. 2014). The analysis focused on nests with hatched nestlings of Small Tree Finches and Green Warbler Finches, excluding Small Ground Finches, Yellow Warblers, and Woodpecker Finches due to insufficient sample sizes (*N* < 9). The post hoc visualization of significant interactions between host density and mean infestation intensity of simultaneously active nests was created using the “interactions” package (version 1.1.5) (Long 2019), which statistically controls for other predictors in the model.

Model (A) (*N* = 102) investigated infestation intensity per nestling as a function of host density and its interaction with mean infestation intensity of simultaneously active nests using a Gaussian error distribution with an identity link function. Nestling number, nest height (Kleindorfer et al. 2021), year, and species identity were included as control predictors to additionally account for potential sources of variation in the response. The full model performed significantly better than the null model, indicating that the inclusion of the interaction term better explains the data, thereby supporting the working hypothesis over the null hypothesis (*χ* ^2^ = 5.313, df = 1, *p* = 0.021).

Model (B) (*N* = 85) assessed the proportion of focal nest activity duration relative to the maximum observed duration (30 days) in relation to host density and its interaction with mean infestation intensity of simultaneously active nests, utilizing beta regression with a logit link function. The 30‐day maximum was chosen for proportion calculations to account for natural variation in fledging timing and to accommodate cases where nests exceeded the typical 28‐day fledging period (Cimadom et al. 2014), as observed in six nests, ensuring a standardized metric for analysis. Nest height, nestling number, species identity, and year were included as control predictors. Nests that were predated or had an unclear fate (e.g., fallen or found destroyed for unknown reasons) were excluded. To ensure the response values remained within a 0–1 range, values of 0 were substituted with 0.0001, and values of 1 were replaced with 0.9999. The full model explained significantly more variation in nest duration than the null model without the interaction term (*χ* ^2^ = 6.647, df = 1, *p* = 0.010).

Model (C) (*N* = 106) investigated the proportion of mature *P. downsi* individuals at the end of nest activity relative to the total larvae count, as a function of host density and its interaction with mean infestation intensity of simultaneously active nests, using beta regression with a logit link function. To maintain the response values within the 0–1 range, values of 0 were replaced with 0.0001, and values of 1 were adjusted to 0.9999. Following this, the values were square root transformed to address under‐dispersion. Nest height, the number of nestlings, species identity, and year were used as control predictors. The full model accounted for variation in the response significantly better than the null model that excluded the interaction between host density and mean infestation intensity of simultaneously active nests (*χ* ^2^ = 5.770, df = 1, *p* = 0.016).

#### 2.6.2 Oviposition During Host Incubation

##### 2.6.2.1 Main Dataset 2020 and 2022

Model (D) (*N* = 84) examined oviposition during host incubation in *P. downsi* (binomial response) in relation to host density and its interaction with mean infestation intensity of simultaneously active nests (test predictors), utilizing a binomial logistic regression with a logit link function. Nest height and year were included as control predictors. Oviposition during host incubation was observed exclusively in Green Warbler Finches; therefore, the analysis was limited to this host species. The full model significantly explained more variation in the response compared to a null model, lacking the interaction term but including all predictors (*χ* ^2^ = 11.770, df = 1, *p* < 0.001).

##### 2.6.2.2 Long‐Term Dataset

In model (E) (*N* = 136), we examined the relationship between oviposition during host incubation in abandoned nests (as a binomial response variable) and two test predictors: mean intensity and prevalence of hatched nests during 2‐week intervals. Besides species identity, the 2‐week time interval, with values between 1 and 8 for each year of the study, was used as a numeric control predictor to account for potential within‐year variation. The number of abandoned nests per time interval was included as a control predictor to control for the effect of abandonment rates on the occurrence or related detection probability of oviposition during host incubation. Study year was included as a random effect, with random slopes initially specified for the categorical year predictor. Due to non‐convergence, random slopes were removed to simplify the model. The model used a binomial error distribution with a logit link function for the incidence data. Testing against a null model without test predictors showed a significant improvement (*χ* ^2^ = 6.552, df = 2, *p* = 0.038), confirming that the full model provided a significantly better fit.

#### 2.6.3 Species Comparison

To compare host density between the Green Warbler Finch and the Small Tree Finch, we used model (F) (*N* = 112) with a Gaussian error distribution and identity link function. This model assessed the effects of species (test predictor), nest height, start of incubation, and year on host density. In comparison to a null model with only control predictors, the full model showed a significant increase in explanatory power for the response variable (*χ* ^2^ = 25.826, df = 1, *p* < 0.001).

## 3 Results

### 3.1 Infestation Patterns

#### 3.1.1 Temporal Trends in Infestation Patterns and Host Distribution Dynamics

**FIGURE 1:**
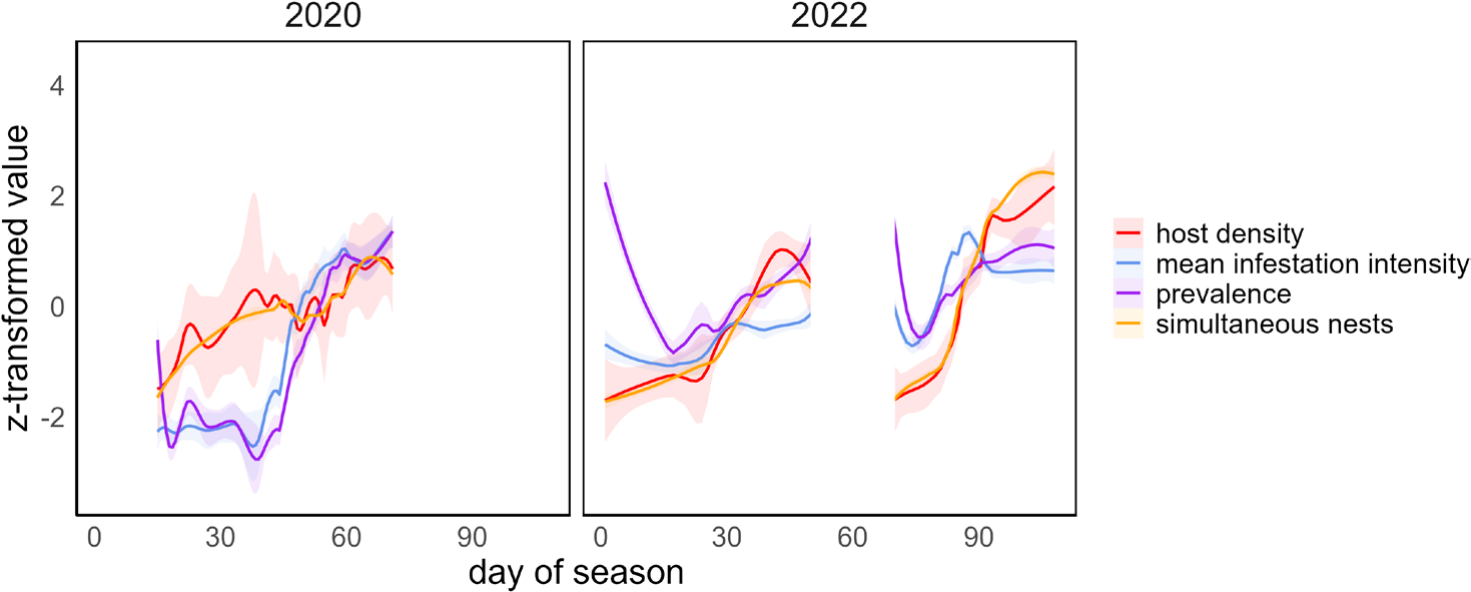
Smoothed trends of host density, mean infestation intensity, prevalence, and simultaneous nests across the 2020 and 2022 study seasons. Each line represents z‐transformed values with 95% confidence intervals (transparent), smoothed using local polynomial regression (span = 0.2). Data are based on focal nests, with host density, mean infestation intensity, and prevalence calculated from simultaneously active nests at the start of incubation of the focal nest. The day of season represents the start of incubation for the focal nest. The white area between days 50 and 70 in 2022 marks a drought‐related interruption of breeding activity, during which no focal nests initiated incubation. *N* = 126.

Host density and the number of simultaneously active nests followed a broad increasing trend throughout the nesting season in both 2020 and 2022, accompanied by a rise in mean infestation intensity and prevalence of simultaneously active nests (Figure 1). At the beginning of the breeding seasons, *P. downsi* prevalence was high in both years. However, as the number of active nests increased, prevalence rapidly decreased early in the season. Mean infestation intensity exhibited a similar pattern, peaking in early 2022 when prevalence was high relative to host density, then leveling off as nest availability increased and *P. downsi* prevalence declined. A comparable situation with high prevalence and intensity occurred in 2022 during a period of low host availability, following a drought‐related interruption of nesting activity of approximately 3 weeks. In contrast, no early‐season mean intensity decrease was evident in 2020. Both initial prevalence (*W* = 2, *p* < 0.001) and mean infestation intensity (*W* = 0, *p* < 0.001) were significantly lower in 2020 compared to 2022 when analyzing only the first 29 days of the seasons. Conversely, host density (*W* = 62, *p* = 0.305) and the number of simultaneous nests (*W* = 57, *p* = 0.486) showed no significant differences between years (*N* = 20).

#### 3.1.2 Host Density and Infestation Patterns in Focal Nests

**FIGURE 2:**
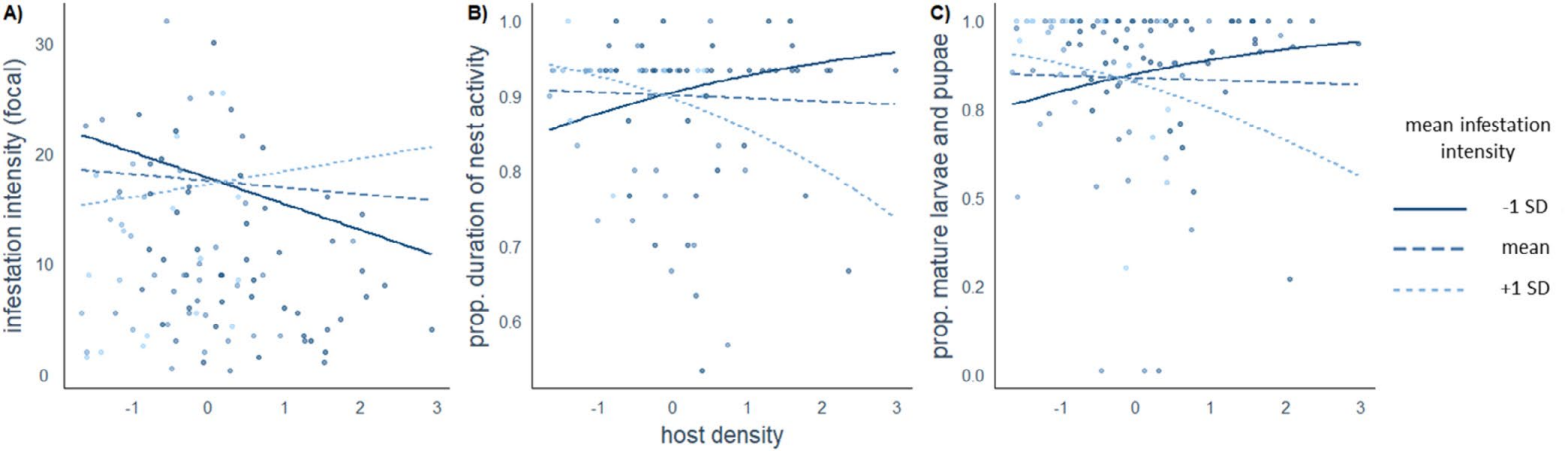
Predicted relationships between per‐nestling infestation intensity (A), nest activity duration (B), proportion of mature larvae and pupae (C), and z‐transformed host density at different levels of mean infestation intensity in simultaneously active nests. Mean infestation intensity is represented by the mean (medium blue, dashed), one standard deviation above the mean (+1 SD, dark blue, solid), and one standard deviation below the mean (−1 SD, light blue, dotted). Point color indicates the corresponding mean infestation intensity for each focal nest.

In model (A), the interaction between host density and mean infestation intensity of simultaneously active nests significantly influenced per‐nestling infestation intensity within focal nests, warranting a follow‐up plot to better understand its effect (Table 2). Post hoc plotting of the interaction term revealed a positive relationship between host density and per‐nestling infestation intensity at low mean infestation intensity of simultaneously active nests (−1 SD), indicating that higher host density corresponded to more *P. downsi* larvae per nestling. In contrast, at high mean infestation intensity of simultaneously active nests (+1 SD), the relationship reversed, suggesting that increased host density resulted in decreased infestation intensity per nestling (Figure 2A). Among the control predictors, the number of nestlings per nest had a significant negative effect on infestation intensity per nestling, whereas nest height and year had no significant effect. Additionally, infestation intensity per nestling was significantly lower in Green Warbler Finches compared to Small Tree Finches.

**TABLE 2:** GLM model results (model A) testing the influence of various predictors on the *Philornis downsi* infestation intensity per nestling of focal nests; reference categories are Small Tree Finch for species and 2020 for year; *N* (observations) = 102.

Predictors	Estimate (95% CI)	SE	*z*	*p*
(Intercept)	1329.411 (−1280.727, 3939.548)	1331.727	0.998	0.318
*z* host density	−0.598 (−1.886, 0.690)	0.657	−0.910	0.363
*z* mean infestation intensity	0.269 (−1.184, 1.722)	0.741	0.363	0.717
*z* nestling number	−3.223 (−4.392, −2.055)	0.596	−5.408	< 0.001***
*z* nest height (focal)	0.164 (−1.077, 1.404)	0.633	0.258	0.796
Year (2022)	−0.649 (−1.940, 0.642)	0.659	−0.985	0.325
Species (Green Warbler Finches)	−8.182 (−11.026, −5.338)	1.451	−5.639	< 0.001***
*z* host density × *z* mean infestation intensity	−1.757 (−3.232, −0.282)	0.752	−2.335	0.020*

*Note:* Asterisks indicate significance: * *p* < 0.05, ** *p* < 0.01, *** *p* < 0.001; bold = significant *p* < 0.05.

Model (B) revealed a significant interaction between host density and mean infestation intensity of simultaneously active nests on the proportional duration of nest activity (Table 3). Plotting of the interaction term showed a negative relationship between host density and nest duration at low mean infestation intensity of simultaneously active nests, indicating that higher host density was linked to reduced nest duration under low‐intensity conditions (−1 SD). Conversely, at high mean infestation intensity of simultaneously active nests (+1 SD), the regression line shifted to a positive slope, indicating that increased host density correlated with a longer nest duration under this condition (Figure 2B). The number of nestlings was significantly positively associated with the duration of nest activity, whereas nest height, year, and species had no significant effect.

**TABLE 3:** GLM model results (model B) testing the influence of various predictors on the proportional duration of nest activity; reference categories are Small Tree Finch for species and 2020 for year; *N* (observations) = 85.

Predictors	Estimate (95% CI)	SE	*z*	*p*
(Intercept)	−98.342 (−557.696, 361.011)	234.368	−0.420	0.675
*z* host density	−0.067 (−0.309, 0.175)	0.124	−0.543	0.587
*z* mean infestation intensity	0.014 (−0.237, 0.265)	0.128	0.107	0.915
*z* nestling number	0.278 (0.078, 0.478)	0.102	2.719	0.007**
*z* nest height focal	0.164 (−0.016, 0.344)	0.092	1.783	0.075
Year	0.050 (−0.178, 0.277)	0.116	0.429	0.668
Species (Green Warbler Finches)	−0.235 (−0.797, 0.326)	0.286	−0.822	0.411
*z* host density × *z* mean infestation intensity	0.311 (0.074, 0.548)	0.121	2.570	0.010*

*Note:* Asterisks indicate significance: * *p* < 0.05, ** *p* < 0.01, *** *p* < 0.001; bold = significant *p* < 0.05.

Results of model (C) showed a significant effect of host density in interaction with mean infestation intensity of simultaneously active nests on the proportion of 3rd instar larvae and pupae relative to the total larvae cohort per nest at the end of nest activity (Table 4). The post hoc visualization of the interaction term (Figure 2C) revealed a contrasting trend in the effect of host density on the reproductive success of *P. downsi*, depending on the mean infestation intensity of simultaneously active nests. At low infestation intensity (−1 SD), the proportion of mature larvae and pupae decreased with increasing host density, whereas at high infestation intensity (+1 SD), the proportion of mature larvae and pupae increased with increasing host density. The control predictors did not show a statistically significant effect on the proportion of mature *P. downsi* individuals.

**TABLE 4:** GLM model results (model C) testing the influence of various predictors on the proportion of 3rd instar larvae and pupae relative to the total larvae cohort per nest; reference categories are Small Tree Finch for species and 2020 for year; *N* (observations) = 106.

Predictors	Estimate (95% CI)	SE	*z*	*p*
(Intercept)	249.117 (−281.645, 779.879)	270.802	0.920	0.358
*z* host density	−0.038 (−0.296, 0.220)	0.132	−0.287	0.774
*z* mean infestation intensity	0.093 (−0.196, 0.382)	0.147	0.629	0.530
*z* nestling number	−0.054 (−0.284, 0.175)	0.117	−0.464	0.643
*z* nest height focal	0.028 (−0.206, 0.262)	0.119	0.236	0.814
Year	−0.122 (−0.385, 0.140)	0.134	−0.914	0.361
Species (wf)	−0.288 (−0.868, 0.293)	0.296	−0.970	0.332
*z* host density × *z* mean infestation intensity	0.372 (0.065, 0.680)	0.157	2.376	0.018*

*Note:* Asterisks indicate significance: * *p* < 0.05, ** *p* < 0.01, *** *p* < 0.001; bold = significant *p* < 0.05.

**FIGURE A1:**
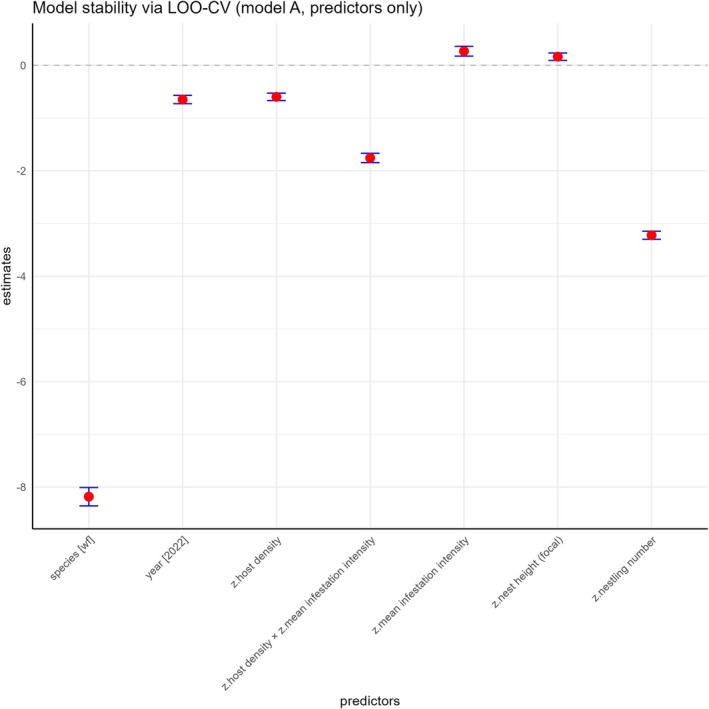
Stability of predictor parameter estimates from a generalized linear mixed‐effects model (Model A) using a leave‐one‐out cross‐validation (LOO‐CV) approach. The blue points represent the mean estimates of each parameter calculated from the LOO‐CV procedure across all subsets of the data. Blue error bars indicate the standard deviation of these estimates, reflecting the variability across the different subsets. The red points represent the original parameter estimates obtained from the model fitted to the full dataset.

**FIGURE A2:**
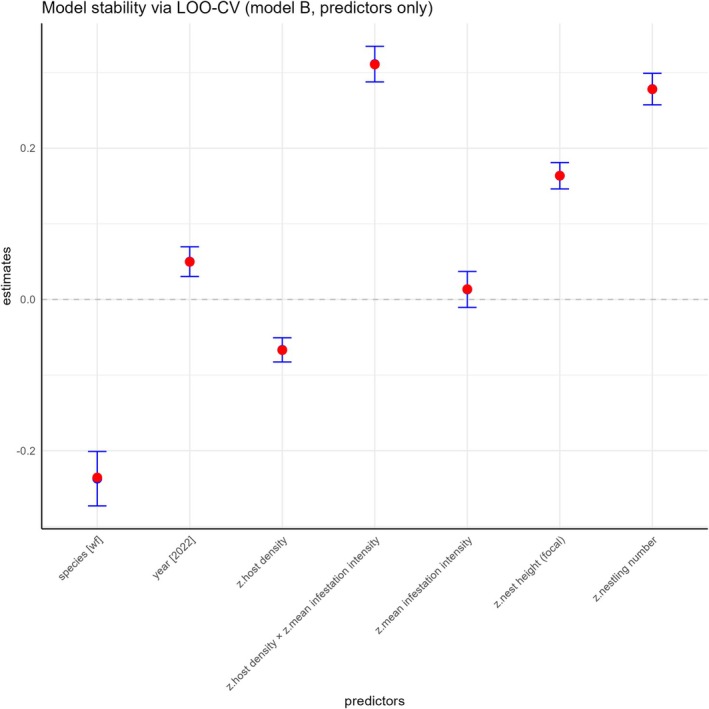
Stability of predictor parameter estimates from a generalized linear mixed‐effects model (Model B) using a leave‐one‐out cross‐validation (LOO‐CV) approach. The blue points represent the mean estimates of each parameter calculated from the LOO‐CV procedure across all subsets of the data. Blue error bars indicate the standard deviation of these estimates, reflecting the variability across the different subsets. The red points represent the original parameter estimates obtained from the model fitted to the full dataset.

**FIGURE A3:**
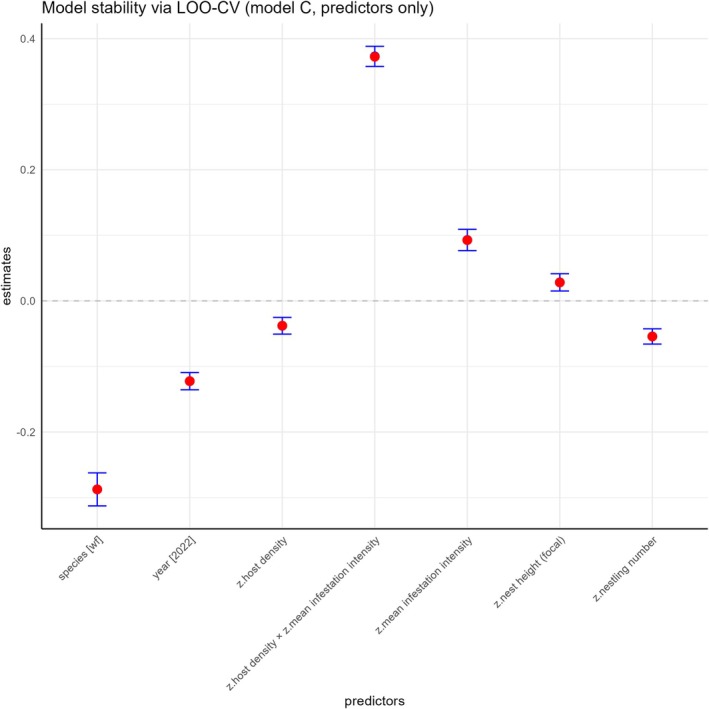
Stability of predictor parameter estimates from a generalized linear mixed‐effects model (Model C) using a leave‐one‐out cross‐validation (LOO‐CV) approach. The blue points represent the mean estimates of each parameter calculated from the LOO‐CV procedure across all subsets of the data. Blue error bars indicate the standard deviation of these estimates, reflecting the variability across the different subsets. The red points represent the original parameter estimates obtained from the model fitted to the full dataset.

**FIGURE A4:**
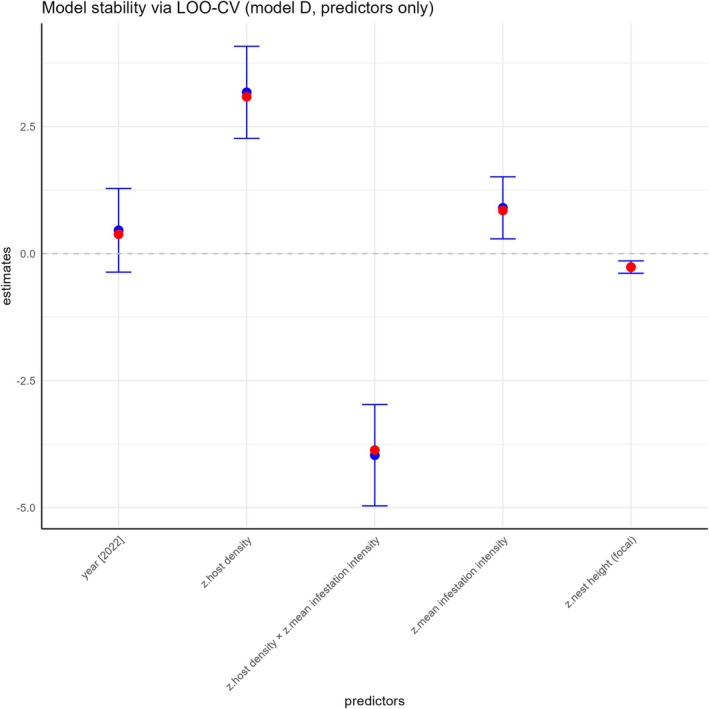
Stability of predictor parameter estimates from a generalized linear mixed‐effects model (Model D) using a leave‐one‐out cross‐validation (LOO‐CV) approach. The blue points represent the mean estimates of each parameter calculated from the LOO‐CV procedure across all subsets of the data. Blue error bars indicate the standard deviation of these estimates, reflecting the variability across the different subsets. The red points represent the original parameter estimates obtained from the model fitted to the full dataset.

**FIGURE A5:**
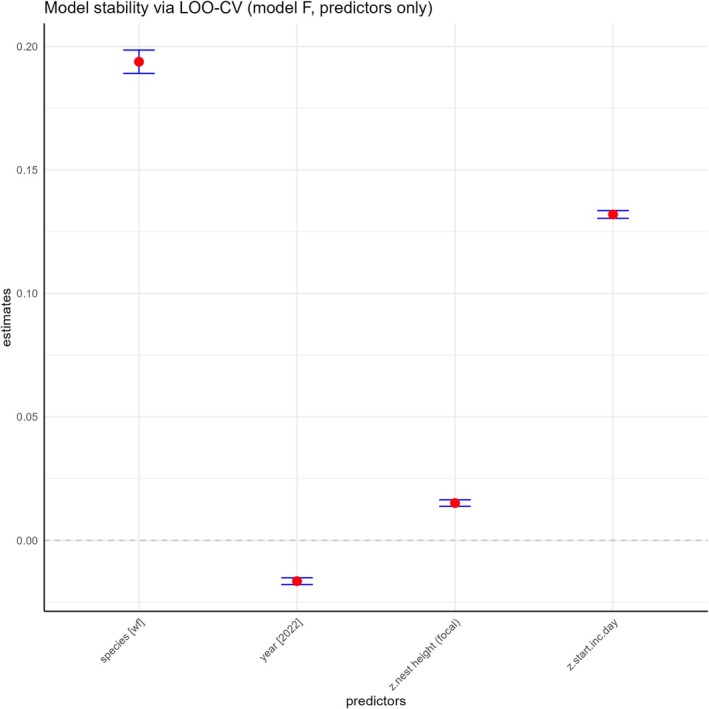
Stability of predictor parameter estimates from a generalized linear mixed‐effects model (Model F) using a leave‐one‐out cross‐validation (LOO‐CV) approach. The blue points represent the mean estimates of each parameter calculated from the LOO‐CV procedure across all subsets of the data. Blue error bars indicate the standard deviation of these estimates, reflecting the variability across the different subsets. The red points represent the original parameter estimates obtained from the model fitted to the full dataset.

LOO‐CV revealed instability in the intercept estimates across Models A–D, suggesting sensitivity to specific data points. However, the direction and strength of key test predictors remained stable across all models (Figures A1, A2, A3, A4, A5).

### 3.2 Oviposition During Host Incubation

#### 3.2.1 Main Dataset 2020 and 2022

**FIGURE 3:**
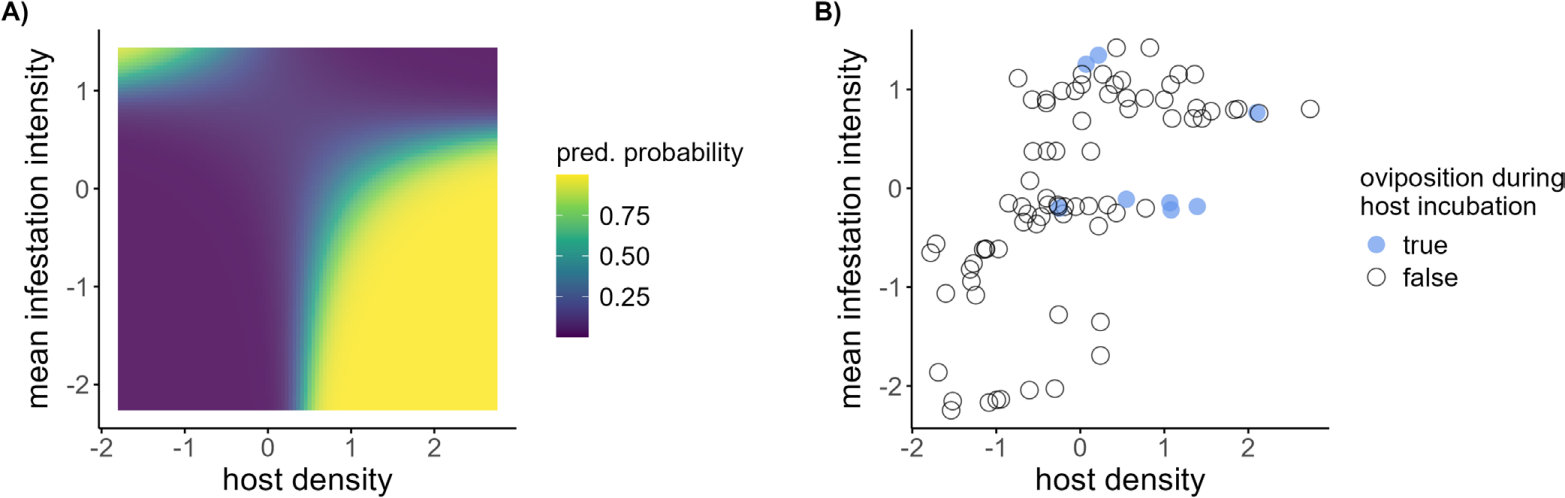
Predicted probability of oviposition during host incubation as a function of z‐transformed host density and mean infestation intensity, with probabilities calculated by model D and shown on the color scale. Average year and nest height values were used for calculations (A). Scatter plot showing the distribution of empirical data within the range of z‐transformed host density and mean infestation intensity of simultaneously active nests, with point style indicating oviposition during host incubation (blue = true, black circle = false) (B).

During 2020 and 2022, 8 out of 84 focal Green Warbler Finch nests were infested during host incubation. Model (D) identified a significant interaction between host density and mean infestation intensity of simultaneously active nests in predicting oviposition during host incubation, while nest height and year had no significant effects (Table 5). Post hoc visualization of the interaction (Figure 3A) showed that host density was positively associated with the probability of oviposition during host incubation. However, this relationship weakened at very high mean infestation intensity. Conversely, low host density and very high mean infestation intensity led to an increased probability of oviposition during incubation.

**TABLE 5:** GLM model results (model D) testing the influence of various predictors on the occurrence of infestation during incubation; reference category is 2020 for year; *N* (observations) = 84.

Predictors	Estimate (95% CI)	SE	*z*	*p*
(Intercept)	−773.65 (−3270.48, 1723.19)	1273.92	−0.61	0.5437
*z* host density	3.09 (0.56, 5.62)	1.29	2.39	0.017*
*z* mean infestation intensity	0.85 (−0.90, 2.60)	0.90	0.95	0.3423
*z* nest height (focal)	−0.27 (−1.23, 0.69)	0.49	−0.55	0.5842
Year	0.38 (−0.85, 1.62)	0.63	0.61	0.5452
*z* host density × *z* mean infestation intensity	−3.87 (−6.87, −0.86)	1.53	−2.52	0.012*

*Note:* Asterisks indicate significance: * *p* < 0.05, ** *p* < 0.01, *** *p* < 0.001; bold = significant *p* < 0.05.

Notably, oviposition during host incubation was not predicted for combinations of host densities and mean infestation intensities at or below average. This aligns with our empirical observations, where no oviposition during host incubation was recorded below a combination of z‐transformed host density of −0.251 SD and mean infestation intensity of −0.219 SD (Figure 3B).

#### 3.2.2 Long‐Term Dataset

Across all study years, infestation by *P. downsi* in nests with hatched nestlings was widespread, with infestation intensities ranging from 1 to 99 larvae per nest. A total of 691 out of 758 nests were infested, reflecting an overall prevalence of 91% across years and host species. Among the 170 nests abandoned during incubation, *P. downsi* larvae were present in 28.8% of nests across years. The prevalence of larvae within incubated nests varied within years, ranging from 9% to 65% overall, 0% to 33% in Small Tree Finches, and 14% to 88% in Green Warbler Finches.

**FIGURE 4:**
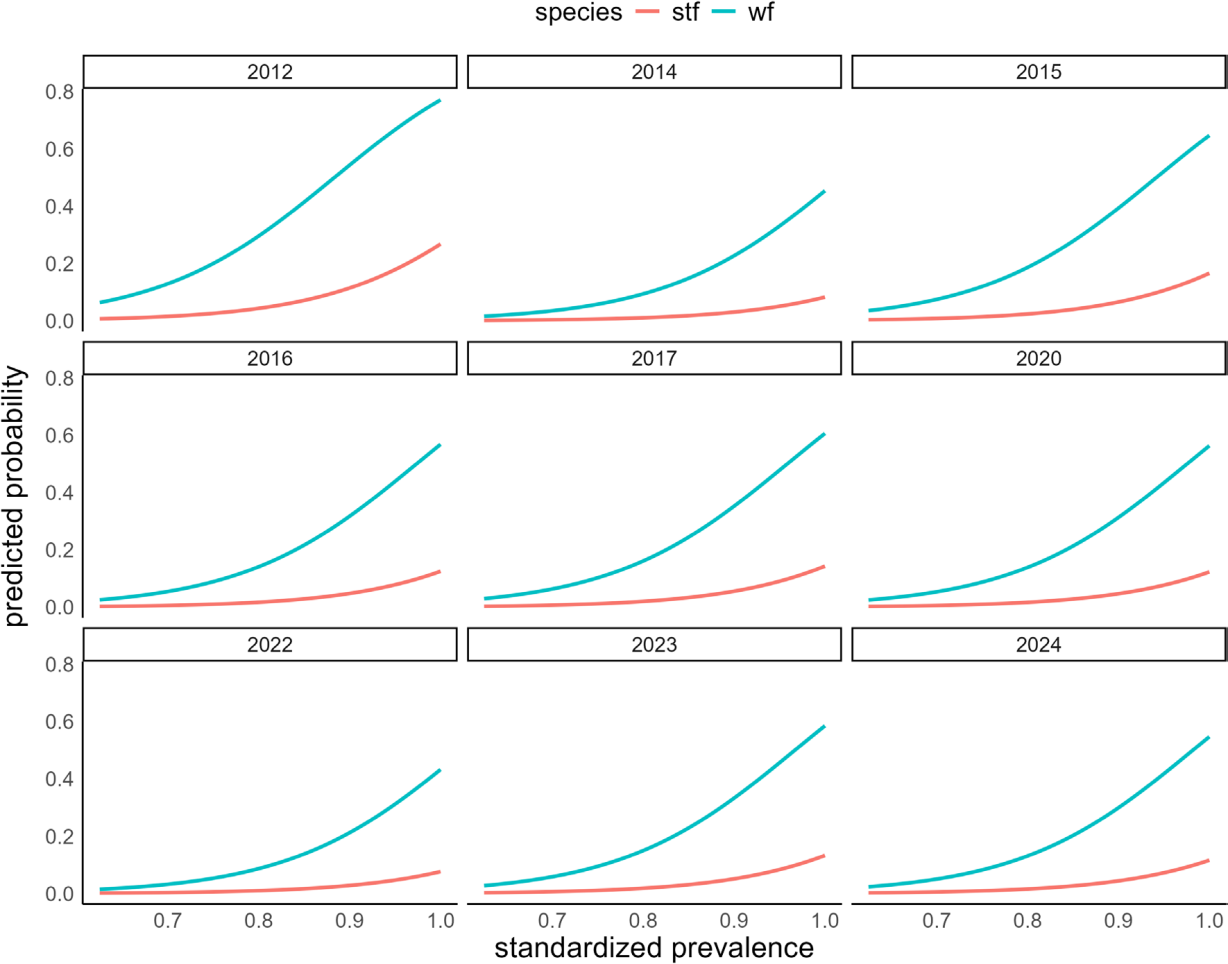
Predicted probability of oviposition during host incubation across standardized prevalence ((prevalence‐mean)/SD), for Green Warbler Finches (wf) = blue line and Small Tree Finches (stf) = red line.

In model (E), oviposition during host incubation significantly increased with an increase in prevalence, while mean infestation intensity showed no significant effect (Table 6; Figure 4). The probability for oviposition during host incubation was significantly higher in Green Warbler Finches compared to Small Tree Finches. The 2‐week interval predictor as well as the number of abandoned nests showed no significant effect on the probability of oviposition during host incubation.

**TABLE 6:** GLMM model results (model E) testing the influence of various predictors on the occurrence of infestation during incubation; reference category is Small Tree Finch for species; *N* (observations) = 136.

Predictors	Estimate (95% CI)	SE	*z*	*p*
(Intercept)	−2.98 (−4.32, −1.65)	0.68	−4.39	< 0.001***
*z* prevalence	0.98 (0.12, 1.84)	0.44	2.22	0.026*
*z* mean infestation intensity	−0.33 (−0.88, 0.22)	0.28	−1.17	0.241
Species (Green Warbler Finch)	2.22 (0.89, 3.54)	0.68	3.28	0.001**
*z* 2‐week interval (numeric)	0.21 (−0.34, 0.77)	0.28	0.75	0.454
*z* number of abandoned nests	−0.09 (−0.83, 0.65)	0.38	−0.24	0.810

*Note:* Asterisks indicate significance: * *p* < 0.05, ** *p* < 0.01, *** *p* < 0.001; bold = significant *p* < 0.05.

### 3.3 Comparison of Host Density Between Species

Model (F) showed a significant difference in host density between two host species, with Green Warbler Finches exhibiting greater densities than Small Tree Finches (*p* < 0.001) (Table 7). Moreover, host density increased during the season (*p* < 0.001) but did not significantly vary between the study years 2020 and 2022. There was no significant relationship between nest height and host density.

**TABLE 7:** GLMM model results (model F) testing the influence of various predictors on host density; reference categories are Small Tree Finch for species and 2020 for year; *N* (observations) = 112.

Predictors	Estimate (95% CI)	SE	*z*	*p*
(Intercept)	33.56 (−30.03, 97.15)	32.44	1.03	0.301
Species (Green Warbler Finch)	0.19 (0.12, 0.26)	0.04	5.42	< 0.001***
*z* nest height	0.02 (−0.01, 0.04)	0.02	1.00	0.319
*z* start of incubation	0.13 (0.10, 0.16)	0.02	8.37	< 0.001***
Year (2022)	−0.02 (−0.05, 0.01)	0.02	−1.03	0.304

*Note:* Asterisks indicate significance: * *p* < 0.05, ** *p* < 0.01, *** *p* < 0.001; bold = significant *p* < 0.05.

### 3.4 Mainland

In mainland Ecuador across all sites and years, a total of 89 nests failed during incubation, representing 19% of all nests monitored. None of these nests were infested by *Philornis* species. Of the 386 nests monitored during the active nest phase, that either fledged or failed after hatching (predated nests excluded), 97 (25%) were found to be infested by one of three co‐occurring *Philornis* species (*P. downsi*, *P. niger* , *P. falsificus*). Total *Philornis* infestation intensity ranged between 1 and 160 larvae per infested nest (mean 29.5). Specifically, *P. downsi* was detected in 54 nests (14%), with *P. downsi* infestation intensity ranging from 1 to 135 larvae per nest (mean 20.2). Among the *P. downsi*‐infested nests, 26 exhibited co‐infestation with other *Philornis* species, and these nests harbored between 1 and 138 additional larvae. The proportion of *P. downsi* larvae in co‐infested nests ranged between 0.7% and 92.6% (mean 43.8%). Forty‐two nests were infested by *Philornis* species other than *P. downsi*, with infestation intensities ranging from 1 to 128 larvae per nest (mean 20.8).

## 4 Discussion

This study highlights the intricate dynamics between intraspecific competition and parasite infestation patterns, shaping oviposition timing in the invasive ectoparasite *P. downsi* within a wild multi‐host system on the Galapagos Islands. We found evidence that in the Galapagos Islands, *P. downsi* oviposited in 29% of Darwin's finch nests that were abandoned during the incubation phase, with yearly prevalence of oviposition during host incubation ranging from 9% to 67%. These results, derived from the long‐term dataset between 2012 and 2024, sharply contrast with observations from mainland Ecuador, the ancestral range of *P. downsi*, where no oviposition during host incubation was observed in this study. *Philornis* larvae are typically parasites of developing nestlings. In the early years following the discovery of *P. downsi* in the Galápagos, larvae appeared to feed solely on nestling hosts (Common, Dudaniec, et al. 2020). However, decades of sampling in Galapagos suggest a shift toward earlier oviposition within the nesting cycle (Kleindorfer et al. 2014) likely driven by increasing intraspecific competition among flies.

In our study, we examine how various ecological factors interact to explain the occurrence of oviposition during host incubation—an aspect not addressed in prior work—as well as the broader implications of this oviposition tactic in *P. downsi*.

### 4.1 Infestation Patterns

#### 4.1.1 Temporal Trends in Infestation Patterns and Host Dynamics

The proportion of host nests experiencing oviposition during host incubation varied with the apparent strength of competition that *P. downsi* was subjected to, as measured by host density and mean infestation intensity as well as parasite prevalence of simultaneously active nests.

During the 2020 and 2022 breeding seasons of Darwin's finches, fluctuations in host availability and fly density indicate phases of intensified intraspecific competition. At the beginning of the breeding seasons, *P. downsi* prevalence was high, likely due to the presence of female flies ready to infest the first nests at elevated rates. Gravid *P. downsi* females can persist in arid habitats with scarce hosts, staying active during breeding and non‐breeding seasons, including prolonged dry periods (Bulgarella et al. 2022; Causton et al. 2019). However, as the nesting season progressed and the number of active nests increased, competition likely decreased, leading to a rapid early‐season decline in prevalence. Mean infestation intensity followed a similar trend, peaking early in 2022 but remaining lower early in the 2020 season, possibly due to more parasite‐free nests. Initial prevalence and mean intensity levels were significantly lower in 2020 than in 2022, despite similar host density and nest numbers. Yearly variations in early‐season *P. downsi* population size may reflect differences in the parasite's reproductive success during the previous bird breeding season, which was drier in 2019 (CDF, unpublished data).

Causton et al. (2019) estimated an egg‐to‐egg generation time ranging from 29 to 56 days, depending on climatic conditions. The emergence of the first generation of flies may mark a shift in the trends of prevalence and mean intensity, as observed in the data. Following the initial phase, both mean *P. downsi* prevalence and intensity increased, coinciding with rising host density and the number of simultaneously active nests. We consider these escalating levels of mean intensity and prevalence to reflect a growing fly population, expanding at a faster rate than the availability of host nests. Although our study did not include the end of the nesting season, where peak competition for limited resources is expected, host availability varied due to unfavorable environmental conditions following a drought‐related decline in nesting activity during the 2022 season, when most oviposition events during host incubation were observed.

#### 4.1.2 Host Density and Infestation Patterns in Focal Nests

We found that the effect of host density varied depending on the mean infestation intensity of simultaneously active nests, impacting infestation levels, nest duration, and parasite reproductive success in nests with hatched nestlings. At low mean infestation intensity, a positive relationship between host density and per‐nestling infestation intensity, coupled with a negative effect on both the duration of nest activity and the proportion of mature larvae and pupae, was observed. Conversely, at high mean infestation intensity of simultaneously active nests, this relationship reversed; increased host density was associated with decreased per‐nestling infestation intensity, an expansion in the duration of nest activity, and an increase in the proportion of mature larvae and pupae relative to the total larvae cohort.

The abundance of mobile avian ectoparasites can increase in nest clusters, as higher host density amplifies detection probabilities, making these aggregations more conspicuous, which not only improves the likelihood for parasites to locate hosts but also allows individual parasites to exploit multiple hosts within these clusters (Brown and Brown 2001; Dudaniec et al. 2010; Veiga et al. 2020). Thus, *P. downsi* flies may be drawn to nest aggregations, where nests located in close proximity to one another experience higher infestation intensities compared to isolated nests (i.e., a fly attraction hypothesis). This can result in shorter periods of nest activity due to larval crowding and ultimately lead to reduced reproductive success for the fly, as indicated by a lower proportion of mature larvae and pupae by the end of nest activity at high‐density areas. While the mechanisms by which *P. downsi* locates its hosts are still not fully understood, evidence suggests that olfactory, visual, or auditory cues, or a combination of these may be involved (Kleindorfer and Dudaniec 2009; Mieles Garcia 2018; Pike et al. 2021).

Parasite mobility is another important factor to consider in this context. Limited information on *P. downsi* dispersal suggests that these flies can migrate between forest patches separated by distances of 600 m, even across harsh environments (Fessl et al. 2018), and high rates of movement are supported by population genetic studies as well (Basnet et al. 2025; Dudaniec et al. 2008; Koop et al. 2021). Therefore, it can be assumed that adult flies are capable of moving throughout the entire study plot in search of suitable hosts. Supporting the fly attraction hypothesis, a study from Santa Cruz Island found that Small Tree Finch nests in aggregations with more than two heterospecific neighbors within a 20 m radius had higher *P. downsi* infestation intensities than solitary nests (Kleindorfer and Dudaniec 2009).

Toward higher levels of mean infestation intensity and host density, a dilution of the spatial effect may occur in two ways: (1) through adult fly numbers, where the likelihood of a gravid female locating a host nest increases for both isolated nests and those within clumps, and (2) through increasing host availability, where a greater number of nests reduces the clustering effect, preventing extreme infestation intensities at any one location.

### 4.2 Oviposition During Host Incubation and Infestation Intensity

In line with observed trends in focal infestation intensity per nestling, the probability of oviposition during host incubation in Green Warbler Finches showed a significant positive association with both host density and mean infestation intensity of simultaneously active nests. However, this effect appeared to weaken at very high mean infestation intensity. The interaction between host density and mean infestation intensity on the occurrence of oviposition should be interpreted with caution due to several limitations. Notably, oviposition during host incubation was infrequent in the study years of 2020 and 2022 (8 cases were observed). Furthermore, certain combinations of predictor variables—such as high host density paired with low mean infestation intensity or high mean infestation intensity with low host density—were rarely or never observed, which introduces uncertainty in the predictions for these conditions. Consequently, conclusions should be limited to the finding that oviposition during host incubation did not occur when both host density and mean infestation intensity of simultaneously active nests were below average but was observed at higher levels of these factors. While it is possible that an interaction between host density and mean infestation intensity may exist under other conditions, the scarcity of data in these specific cases prevents a definitive identification of this relationship, highlighting the need for further research to explore these effects more thoroughly.

Our results suggest that not only host density plays a significant role in shaping intraspecific competition in *P. downsi*, as already shown by Kleindorfer and Dudaniec (2009), but also the interaction with mean infestation intensity, as reflected in infestation patterns at individual nests. Our findings reveal a previously undocumented complexity in *P. downsi* infestation dynamics, showing a reversal of infestation patterns at the per‐nest level under high intraspecific competition: with increasing mean infestation intensity in simultaneously active nests, a clear turning point in infestation patterns occurred at the average host density. At this threshold, we observed a marked increase in the predicted probability of oviposition during host incubation. While the correlational nature of our data prevents a complete understanding of the complex dynamics between predictors and their causal relationships, further studies, including spatial models or experimental approaches, are necessary to fully disentangle these interactions. It is likely that at low to moderate levels of mean infestation intensity in simultaneously active nests, spatial effects play a dominant role in shaping fly competition. However, at very high levels of mean infestation intensity, spatial clustering likely has a reduced role.

Analysis of the long‐term dataset, which did not include host availability, revealed that prevalence was a significant predictor of oviposition in incubated host nests, likely capturing the broader pattern of *P. downsi* competition. Although high prevalence levels at the onset of the breeding season are consistent with competition bottlenecks, suggesting high fly abundance relative to available nests, oviposition during host incubation was not observed during this period. This may be due to an increase in host availability and a corresponding decline in competition at the beginning of the nesting season. In contrast, when the number of simultaneous nests had already peaked or begun to decline, we observed oviposition during host incubation, likely due to heightened fly competition.

Nests can only be inspected for fly presence once activity has ceased. While the detection of oviposition during host incubation is closely tied to nest abandonment during incubation, no significant difference was found in abandonment rates between early‐infested and non‐infested nests at the time of failure. This suggests that detection probability was not influenced by nest abandonment timing, ruling out any potential bias in the observed timing of oviposition during host incubation. However, the potential impact of *P. downsi* infestation on nest abandonment during incubation cannot be excluded. Increased energy demands or irritation for brooding female birds may disrupt incubation, leading to prolonged embryo development or death (Fessl et al. 2018; Knutie et al. 2016; Koop et al. 2013). Opportunistic nest abandonment may also help reduce reproductive costs when nests are parasitized. Given the long lifespan of Galapagos landbirds, life‐history trade‐offs likely favor future reproductive potential over increasing current reproductive effort (Pike et al. 2023).

### 4.3 Oviposition During Host Incubation: Larval Feeding Strategies and Associated Trade‐Offs

The aforementioned dilution effect alone does not fully explain the observed reversal in the *P. downsi* infestation patterns, where increased host density and high mean infestation intensity are associated with reduced per‐nestling infestation intensity. This reduction, occurring alongside the earlier shift in the fly's life cycle under elevated intraspecific competition, suggests that decreased infestation intensity may be associated with the costs or adaptive responses related to suboptimal oviposition timing. Two potential feeding strategies are associated with oviposition during host incubation: (1) feeding on adult birds, supported by immunological data (Huber et al. 2010; Koop et al. 2013) and our data showing the presence of mature larvae and pupae in nests containing host eggs, and (2) “waiting” for the presence of nestlings and feeding exclusively on them (Sage et al. 2018). At incubation temperatures (~33°C), larvae can survive for up to 5 days after oviposition, allowing *P. downsi* to potentially rely on nestlings even if eggs are laid earlier in the incubation period, without requiring parental feeding (Sage et al. 2018). It is likely that both feeding strategies are employed, with their relative importance contingent upon the timing of oviposition. If oviposition occurs primarily toward the end of incubation, larvae are more likely to endure until nestlings hatch without relying on a food source. In contrast, earlier oviposition would increase larvae's dependence on parental birds for sustenance. Even in this case, however, we assume that larvae would feed on nestlings once they hatch.

Potential trade‐offs for the parasite associated with infestation during host incubation, which may contribute to the observed reduction in infestation intensity, are influenced by the larval feeding strategy. These potential trade‐offs include an increased risk of predation by adult birds, selective nest abandonment, reduced host quality and food accessibility in the case of feeding on brooding females, depletion of finite larval resources in the case of larvae awaiting hatchlings, unfavorable microclimatic conditions, and heightened virulence leading to intensified competition once nestlings hatch (Clayton et al. 2010; Dube et al. 2018; Fitze et al. 2004; Kleindorfer et al. 2014; Pike et al. 2024, 2021; Sage et al. 2018). Such factors may directly impact egg and larval survival, or be mitigated by adaptive strategies such as reduced egg deposition or avoidance of re‐infestation during the early stages of nesting (Koppik et al. 2014). Furthermore, parasite‐induced immunological responses may contribute to the observed variation in infestation intensities. Previous studies demonstrated that incubating female birds exhibited elevated levels of *P. downsi*‐binding antibodies, which were associated with reduced parasite intensities in their nests following exposure to *P. downsi* (Huber et al. 2010; Koop et al. 2013). Maternal antibody transfer during egg‐laying (Buechler et al. 2002) may also play a role in reducing infestation intensity in subsequent nesting attempts. Regardless of the underlying mechanisms, reduced infestation intensity appears to benefit the parasite by likely extending the duration of nest activity and enhancing individual larval survival through decreased crowding, as evidenced by the higher proportion of mature larvae and pupae observed at the end of nest activity.

### 4.4 Species‐Specific Differences in Oviposition Timing and Infestation Patterns

Oviposition during host incubation was significantly more common in Green Warbler Finches compared to Small Tree Finches. The Green Warbler Finch, the most abundant species in the study plot, occupies smaller territories (13.2 m radius) than Small Tree Finches (22.1 m radius) (calculated from density estimates in Dvorak et al. 2012). This results in higher nest densities for Green Warbler Finches. Given the positive relationship between host density and oviposition during host incubation, we suggest that nest distribution may contribute to the observed species differences in oviposition timing.

Cimadom and Tebbich (2021) found lower parasite intensity and a higher proportion of mature larvae and pupae in Green Warbler Finch nests compared to Small Tree Finch nests during the early nestling phase, focusing on nests with nestlings aged 5 days or younger. Additionally, Green Warbler Finches had higher nesting success later in the season (Cimadom et al. 2014). This aligns with the infestation patterns and competition dynamics observed in our study across varying host densities. As noted earlier, we propose that the observed shift in the parasite's life cycle may be linked to lower per‐nestling infestation intensity. Consequently, differences in *P. downsi* infestation intensity and reproductive success, as well as seasonal variation in host nesting success, may arise from the higher likelihood of oviposition during host incubation in Green Warbler Finches, potentially driven by spatial effects. Moreover, long‐term shifts in infestation intensity between Green Warbler Finch and Small Tree Finch were observed during the past decade of research, with decreasing intensity in Green Warbler Finches and increasing intensity in Small Tree Finches with nestlings > 6 days of age (Cimadom and Tebbich 2021). This temporal shift may also reflect changes in *P. downsi* oviposition timing, aligning with the period when oviposition events were first observed on Santa Cruz in 2012 (Cimadom et al. 2016).

### 4.5 Mainland Ecuador

In ancestral continental populations on mainland Ecuador, no oviposition during host incubation was detected, suggesting that oviposition during host incubation is either absent or much less common compared to the Galapagos Islands. Although multiple *Philornis* species co‐occur, competition for host resources is much lower than in the Galapagos, where only *P. downsi* exists and infestation prevalence is substantially higher. The host–parasite co‐evolutionary process on the mainland is likely more advanced due to its longer history, potentially having reached an equilibrium where evolved host defenses result in lower virulence, reduced fly abundance, and lower infestation prevalence (but see Knutie et al. 2017). Additionally, the larger gene pool may further strengthen these defenses. In this context, the need for extreme strategies, such as oviposition during host incubation, may be less pronounced on the continent. As a result, we may be observing a rapid divergence in parasite behavior between the Galapagos and mainland Ecuador, likely driven by the higher competition among flies in Galapagos. One possible explanation for this shift in infestation timing is natural selection, where genetic variation in oviposition timing is linked to reproductive success, influenced by host abundance and parasite intensity. Alternatively, oviposition during host incubation may result from behavioral flexibility or epigenetic processes, where larval conditions influence reproductive behavior in adult flies. The current competition‐dependent strategy points toward the latter explanation, but future genetic studies are necessary to elucidate the underlying mechanisms in the earlier shift in the life cycle of *P. downsi*.

## 5 Conclusions

This study reveals how intraspecific competition in the invasive parasitic fly *P. downsi* drives an advancement in oviposition timing, potentially shifting its feeding target to brooding adult females—or alternatively, causing the larvae to wait for the host nestlings to hatch. This adaptation helps the parasite overcome larval crowding and resource depletion but carries trade‐offs. Thus, oviposition during host incubation likely occurs only under high competition, where the benefits outweigh the costs. Crucially, the observed shift in infestation timing raises significant questions about its impact on host fitness, as increased virulence associated with earlier oviposition timing may further push host populations to the brink of extinction. On the other hand, our data suggest that earlier oviposition may be associated with a reduced infestation intensity per nestling and a prolonged duration of nest activity, while also potentially allowing hosts to benefit from maternal antibody transfer in following generations. This dynamic could lead to a situation where heightened intraspecific competition forces the evolving host–parasite system toward a more balanced state. The shift in timing observed in this study may be a pivotal factor in shaping the future dynamics of this fragile interaction. Given these findings, continuation of long‐term monitoring of this intricate host–parasite system in the Galapagos Islands is critical. However, considering the current situation, with vulnerable host species already experiencing severe population declines, there is an urgent need to expedite research into effective control measures and to implement them to safeguard the archipelago's unique avifauna. It remains to be determined how flies decide whether to repeatedly infest a nest or adjust their timing in response to intraspecific competition, as well as how *P. downsi* utilizes its sensory abilities to perceive its environment and assess the infestation status of an encountered host nest. Genetic studies and experimental approaches could provide valuable insights into host–parasite dynamics, offering crucial knowledge to inform management strategies not only for *P. downsi* but also for other *Philornis* species that impact endangered bird populations (Bulgarella et al. 2019).

## Author Contributions

**Barbara Kofler:** data curation (lead), formal analysis (lead), investigation (lead), methodology (equal), visualization (lead), writing – original draft (lead). **Denis Mosquera:** data curation (supporting), investigation (supporting), writing – review and editing (equal). **George E. Heimpel:** funding acquisition (supporting), resources (supporting), writing – review and editing (equal). **Heinz Richner:** conceptualization (equal), methodology (equal), writing – review and editing (equal). **Sabine Tebbich:** conceptualization (equal), investigation (supporting), methodology (equal), project administration (lead), resources (lead), supervision (lead), writing – review and editing (equal).

## Conflicts of Interest

The authors declare no conflicts of interest.

## Data Availability

The datasets analyzed for this study can be found on the University of Vienna data repository PHAIDRA: https://phaidra.univie.ac.at/o:2126922, https://phaidra.univie.ac.at/o:2126923, and https://phaidra.univie.ac.at/o:2126924.
